# Building an open-source system test generation tool: lessons learned and empirical analyses with EvoMaster

**DOI:** 10.1007/s11219-023-09620-w

**Published:** 2023-03-06

**Authors:** Andrea Arcuri, Man Zhang, Asma Belhadi, Bogdan Marculescu, Amid Golmohammadi, Juan Pablo Galeotti, Susruthan Seran

**Affiliations:** 1 Kristiania University College, Oslo, Norway; 2 University of Buenos Aires and CONICET, Buenos Aires, Argentina; 3 Oslo Metropolitan University, Oslo, Norway

**Keywords:** Software testing, SBST, Tool, Fuzzing, Experimentation

## Abstract

Research in software testing often involves the development of software prototypes. Like any piece of software, there are challenges in the development, use and verification of such tools. However, some challenges are rather specific to this problem domain. For example, often these tools are developed by PhD students straight out of bachelor/master degrees, possibly lacking any industrial experience in software development. Prototype tools are used to carry out empirical studies, possibly studying different parameters of novel designed algorithms. Software scaffolding is needed to run large sets of experiments efficiently. Furthermore, when using AI-based techniques like evolutionary algorithms, care needs to be taken to deal with their randomness, which further complicates their verification. The aforementioned represent some of the challenges we have identified for this domain. In this paper, we report on our experience in building the open-source EvoMaster tool, which aims at system-level test case generation for enterprise applications. Many of the challenges we faced would be common to any researcher needing to build software testing tool prototypes. Therefore, one goal is that our shared experience here will boost the research community, by providing concrete solutions to many development challenges in the building of such kind of research prototypes. Ultimately, this will lead to increase the impact of scientific research on industrial practice.

## Introduction

Automated test case generation is a topic that has been widely studied in the research community (Bertolino, 2007). Throughout the decades, thousands of scientific articles have been written on the subject. Different techniques have been investigated to solve this scientific problem, where the use of *Evolutionary Algorithms* has been one of the most effective solutions (Harman et al., 2012), as well as Dynamic Symbolic Execution (Baldoni et al., 2018).

To investigate different research questions on this scientific topic, tool prototypes have been developed to carry out empirical studies. Some of these tools were just “throw-away” prototypes, meant to be used to answer the research questions for just a single research study. Others were developed and maintained throughout several studies, with a level of engineering effort that would enable even practitioners to use those tools. There are several of these kinds of tools, and a few of them are open-source. A short, non-exhaustive list comprises for example AFL^1^ for security testing of data parsers, KLEE^2^ for dynamic symbolic execution (Cadar et al., 2008), EvoSuite^3^ for evolutionary unit testing (Fraser & Arcuri, 2011), and Randoop^4^ for random testing (Pacheco & Ernst, 2007).

When building a test generation tool to be used in several scientific studies, the complexity of the tool itself increases significantly. In terms of size and complexity, it can become not so different from many pieces of software built in industry. Employing best practices from industrial software development becomes hence essential. For example, the use of version control systems (e.g., Git) and continuous integration is strongly recommended. However, when dealing with the development of test case generator tools (and, more in general, software engineering research prototypes), there are several specific challenges and common problems that researchers need to address. Failing to do so might lead to excessive waste of human resources (time and effort), with prototypes that become hard to maintain and extend for new studies. Furthermore, if such prototypes are faulty, conclusions drawn from empirical studies could be totally wrong, significantly harming the progress of scientific research.

It is quite common that prototypes in software engineering research are built by young PhD students and postdoc researchers. They might or might not have previous industrial experience, or general exposure to the development of large/complex systems. Outstanding senior researchers can provide essential scientific supervision, but might lack professional software development experience, or they might simply not deal with actual software development any more (similarly to managers in industry). Furthermore, in many scientific institutions around the world, excellence is evaluated based on the number of published scientific articles, and not “impact” of such work (e.g., benefits to software engineering industrial practice). On the one hand, new prototypes are built from scratch by lone PhD students as research tends to be directed toward unexplored topics, instead of focusing in depth on a specific scientific problem, and solve it to an extent that will impact industrial practice (Arcuri, 2018b). This latter might require a long-term investment in terms of software prototype development, longer than the duration of typical job contracts in academia. On the other hand, PhD students and postdoc researchers in software engineering are not software developer consultants. Their career progress in academia will be usually based on pedagogic skills and scientific output, not on their software engineering skills. Spending too much time on software engineering tasks instead of concentrating on scientific output might be detrimental for their academic career.

Achieving the right balance, or even being able to define what a “right balance” should be in this context, is likely way beyond what can be provided in this article. Regardless, improving software development practices in software engineering research would be beneficial. Even when addressing different topics in software engineering research, many software tasks will be the same. Learning from existing success stories, with their low-level technical details, might bootstrap new research efforts, and avoid “re-inventing the wheel” on already solved technical problems.

For example, when carrying out empirical studies, it is common to deal with different *parameters*. Those could be settings of the algorithms (e.g., population size and crossover rate in a Genetic Algorithm) which might need to be tuned (Arcuri & Fraser, 2013), as well as activation of new algorithm improvements. This is needed when evaluating if a new algorithmic variant is indeed better than the current state of the art. Given a tool prototype and a possible algorithm variant *X*, it would be important to run the tool with and without *X*. If this is controlled with a boolean parameter, then it can be treated as any of the other parameters. With the growth of a tool from study to study, we could end up with a large number of parameters. Efficiently dealing with such a large number of parameters can significantly save time, especially when the running of the experiments is tailored to use these parameters (e.g., software scaffolding/scripts to run experiments on clusters of computers).

Another common issue is that many techniques to address software engineering problems rely on *randomized* algorithms. Not only does this pose challenges on the analysis of empirical experiments (Arcuri & Briand, 2014), but also on the verification of the tool prototypes themselves. For example, considering a test case generation tool, how can we be sure that an achieved low coverage on a test subject is not due to faults in the tool itself? And, when there is a crash, how to reliably reproduce it to help debug its cause?

These examples are rather general, but there are also very specific issues in more narrow domains. For example, when dealing with the testing of enterprise/web applications, how to deal with the resetting of database state to make test cases independent? Simply restarting the database at each test case execution would be a huge performance hit for any test case generation tool.

Since 2016, we have been developing the EvoMaster open-source tool^5^. EvoMaster is a search-based tool that aims at system test generation for web services, such as RESTful and GraphQL APIs. At the time of this writing, seven people have actively contributed to its development^6^ (i.e., all the authors of this paper), which now spans over more than 200 thousand lines of code (just for the tool; if also including all files used in the test cases, it is several hundreds of thousands of lines). The tool has been used in 18 peer-reviewed published studies so far (plus several more either in press, under review or in writing)  (Arcuri, 2017a, b, 2018a, c, 2019, 2020; Arcuri & Galeotti, 2019, 2020a, b, 2021a; Arcuri et al., 2021; Belhadi et al., 2022; Marculescu et al., 2022; Zhang & Arcuri, 2021a, b; Zhang et al., 2019, 2021; 2022). As it is open-source, other research groups have been using and extending EvoMaster for their research work (e.g., Sahin & Akay, 2021; Stallenberg et al., 2021), independently from us.

In this paper, we report on our experience in developing such a tool for scientific research in software testing. We will mainly focus on technical details that would be of interest for other researchers building research prototypes. Some of the discussions will be specific for software testing, whereas others will be more general for software engineering at large. The main contribution of this paper is to provide useful technical know-how for researchers, to help bootstrapping new or existing engineering effort in tool prototype development. Ultimately, the goal is to help practitioners to benefit from software engineering research.

This paper is organized as follows. Section 2 discusses related work. Section 3 provides a high-level overview of the EvoMaster tool, to better understand the discussions in the remaining of the paper. Section 4 presents how we deal with parameters, i.e., the different settings under which EvoMaster can be run. Section 5 shows how we run experiments when evaluating new techniques, and how their results are automatically analyzed with the appropriate statistical tests. In Sect. 6 we discuss how we write End-to-End tests to verify EvoMaster itself. Section 7 goes into some technical details that are specific for web/enterprise applications. Section 8 lists some general software engineering practices that we found particularly useful during its development, whereas Sect. 9 reports on our experience in dealing with open-sourcing EvoMaster. How our lessons learned can be generalized is discussed in Sect. 10. Finally, Sect. 11 concludes the paper.

## Related work

In the literature, there has been work reporting on the experience of introducing test data generation tools in industry (Brunetto et al., 2021), as well as reporting the experience of commercializing a bug-finding research tool (Bessey et al., 2010). However, to the best of our knowledge, no work in the literature has aimed at providing the experience of building such tools, with the main aim of sharing concrete technical solutions to help new tool prototype development efforts by other researchers. This is a novel contribution provided in this paper.

In the software engineering research literature, several prototypes are developed and extended each year. It is not uncommon to have special *demo tracks* at the major software engineering conferences. For example, looking at their most recent (at the time of this writing) 2021 editions, this was the case for the International Conference on Software Engineering (ICSE) (e.g., Haryono et al., 2021; Vadlamani et al., 2021; Wang et al., 2021), the ACM Joint European Software Engineering Conference and Symposium on the Foundations of Software Engineering (ESEC/FSE) (e.g., Ahmed et al., 2021; Heumüller et al., 2021; Horlings & Jongmans, 2021), the IEEE/ACM International Conference on Automated Software Engineering (ASE) (e.g., Brida et al., 2021; Pham et al., 2021, Xie et al., 2021), and the ACM SIGSOFT International Symposium on Software Testing and Analysis (ISSTA) (e.g., Hou et al., 2021; Natella & Pham, 2021; Ren et al., 2021). However, those are usually short 4-page papers, mainly meant to demonstrate the usefulness of these tools, with not much space available to share concrete technical solutions which can be re-used.

Nowadays, few conferences also have *Artifact Evaluation* tracks, where the authors of accepted research papers are encouraged to submit the artifacts used in the experiments. However, typically there is a lack of info about how these tools are built.

Building software prototypes is a common occurrence in scientific research, and not just in software engineering. A common practice is to release scientific software as *open-source*, as it helps scientific replicability (among the many benefits). However, the development of scientific software is often not properly incentivized in academia. To help tackle this issue, the open-access, peer-reviewed *Journal of Open Source Software* (JOSS) publishes work based on scientific open-source projects, like for example EvoMaster itself (Arcuri et al., 2021). However, the actual published papers are short, up to 1000 words, which does not leave much space to discuss technical details and share lessons learned.

Novel techniques are typically compared with the current state of the art. Often, this is represented by existing tools developed and released by other research groups. “The underlying assumption is that existing systems correctly represent the techniques they implement” (Rizzi et al., 2016). But such assumption is not always valid. For example, Rizzi et al. (2016) introduced six engineering, non-“new” improvements to the popular tool KLEE. This led to drastic performance increases, questioning some existing work in the literature that compared with KLEE. Unfortunately, the academic system puts more emphasis on and incentivizes publishing “novel” techniques rather than providing known software engineering improvements, even if those can have much stronger effects on performance: “robust advances must be built on robust foundations” (Rizzi et al., 2016).

The closest type of work to this article is what gets presented at the IEEE International Conference on Software Testing, Verification and Validation (ICST). Like the other main software engineering conferences (e.g., the aforementioned ICSE, FSE, ASE and ISSTA), it has a *demo track*. However, it also has a *tool track*. Where the demo track is equivalent to the demo tracks in the other conferences, the tool track provides more space (typically between 6 and 10 pages, depending on the year). This enables the space to discuss technical details like architectural choices, which might help to share useful technical information. Note, this might be possible in regular research papers as well (especially in journals, due to more pages available). But such information might be difficult to find out and gather, as spread out among the scientific content which is the main contribution of those articles.

**Table 1:** Descriptive statistics of the tool track at ICST, in the last 5 years

Year	#Pages	#Articles	#Open-Source	Articles
2022	10	6	6	Gotz et al. (2022), Tzoref-Brill et al. (2022), Herlim et al. (2022), Paduraru et al. (2022), Yavuz (2022), Leotta et al. (2022)
2021	6	8	8	Slob and Jongmans (2021), Paduraru et al. (2021), Olianas et al. (2021), Slob and Jongmans (2021), Weiss and Tonella (2021), Arcaini et al. (2021), Romdhana et al. (2021), Bures et al. (2021), Cox (2021)
2020	6	6	6	Bertolino et al. (2020), Bures et al. (2020), He et al. (2020), Sartaj et al. (2020), Udeshi et al. (2020), Pham et al. (2020)
2019	8	5	1	Borges and Zeller (2019), Khaireddine et al. (2019), Musco et al. (2019), Jendele et al. (2019), Paiva et al. (2019)
2018	6	5	3	Arcuri (2018a), Sullivan et al. (2018), Ribeiro et al. (2018), Rapos and Cordy (2018), Hodován and Kiss (2018)
Total		30	24

**Table 2:** Details of the open-source tools published at ICST 2018-2022, in chronological order They are all hosted on GitHub. We report the year of the ICST *Edition* in which the tool was published; the years of the *First* and *Last* commits in tools’ source code Git repositories; the number of *Authors* of the tool papers, as well as the number of code *Contributors* and number of *Stars* the Git repositories have on GitHub. Statistics were collected in March 2022

Name	Edition	First	Last	#Authors	#Contr.	#Stars
Jaguar (Ribeiro et al., 2018)	2018	2014	2019	5	5	18
EvoMaster (Arcuri, 2018a)	2018	2016	2022	1	11	227
Fuzzinator (Hodován & Kiss, 2018)	2018	2016	2022	2	6	184
SmokeOut (Musco et al., 2019)	2019	2018	2018	3	2	0
AFLNet (Pham et al., 2020)	2020	2019	2022	3	28	514
Callisto (Udeshi et al., 2020)	2020	2019	2020	3	1	1
CDST (Sartaj et al., 2020)	2020	2019	2021	3	1	2
ct-fuzz (He et al., 2020)	2020	2019	2020	3	2	6
Groucho (Bertolino et al., 2020)	2020	2019	2021	4	5	2
Prut4J (Slob & Jongmans, 2021)	2021	2019	2021	2	2	0
diffcov (Cox, 2021)	2021	2020	2020	1	1	5
COSMO (Romdhana et al., 2021)	2021	2020	2021	5	2	8
ROBY (Arcaini et al., 2021)	2021	2020	2021	4	4	1
Uncertainty-Wizard (Weiss & Tonella, 2021)	2021	2021	2022	2	1	29
STILE (Olianas et al., 2021)	2021	2020	2020	5	1	0
RiverFuzzRL (Paduraru et al., 2021)	2021	2015	2021	3	12	39
Assessor (Leotta et al., 2022)	2022	2021	2021	3	1	0
SIFT (Yavuz, 2022)	2022	2022	2022	1	1	0
RiverGame (Paduraru et al., 2022)	2022	2020	2022	3	3	0
CITRUS (Herlim et al., 2022)	2022	2021	2021	3	2	0
Tackle-Test (Tzoref-Brill et al., 2022)	2022	2021	2022	5	4/7	23/30
Whisker (Gotz et al., 2022)	2022	2018	2022	3	12	11

Let us analyze in more detail the ICST tool track in the last 5 years. Table 1 provides some general descriptive statistics on these 5 editions of ICST. A total of 30 articles were published. The large majority of these articles (24 out of 30) report on tools that are open-source. In two cases (i.e., for AUnit (Sullivan et al., 2018) and TIARA (Borges & Zeller, 2019)), although they are not open-source, executables are provided online. Table 2 shows more details on 22 of them. Two were excluded: TbUIS (Bures et al., 2020), because it is a testbed/benchmark, and it is not hosted on GitHub (which makes the collections of some statistics in Table 2 not possible); and PatrIoT (Bures et al., 2021), as it was rather unclear to determine which GitHub repositories store the software discussed in that article.

From these 22 tools reported in Table 2, few interesting observations can be made. Only 8 out of 22 tools have still any development in the first months of 2022 (up to late March, the time this data was collected). About one-third of these 22 tools (7 to be precise) have been under development for more than 2 years (based on the actual months of first and last commit, which is not reported in Table 1). And only 4 of these tools have been under development for more than 5 years. There are 7 tools that have been implemented by a single developer, although the number of authors can be up to 5. Only 2 of these tools (i.e., with a single developer) have any code contribution in 2022. The number of GitHub *stars* can be considered an indirect metric of interest expressed by practitioners^7^: only 3 tools (i.e., AFLNet (Pham et al., 2020), EvoMaster (Arcuri, 2018a) and Fuzzinator (Hodován & Kiss, 2018)) have more than 100 stars (although, of course, this also strongly depends on the age of the projects).

This data confirms our initial conjectures: many tools developed in academia have short lifespan, often developed by a single person, and not by groups for long periods of time. However, there are some exceptions, which can be used as useful inspiration and learning sources.

## Background: EvoMaster tool

The EvoMaster tool has been under development and open-source since 2016 (Arcuri, 2017b, 2018a). It aims at generating system-level test cases for Web APIs, like REST and GraphQL. Internally, it uses evolutionary algorithms like MIO (Arcuri, 2018c) to evolve test cases, using white-box heuristics to maximize code coverage and fault finding.

When generating tests for a Web API there are many decisions to make, like setting up query parameters and body payloads as JSON objects. All this information is present in the *schema* of the API (e.g., in OpenAPI format for REST APIs), which needs to be fetched and analyzed by the fuzzer. This creates a huge search space of possible test cases, where EvoMaster uses evolutionary algorithms to efficiently explore it.

Internally, EvoMaster is divided into two main components: a *core* process, and a *driver* library (one for each supported programming environment, like JVM and NodeJS). The *core* process is written in Kotlin, and it contains all the code related to the evolutionary algorithms, computation of fitness function (e.g., how to make HTTP calls for REST and GraphQL APIs), generation of source code (e.g., in JUnit format) for the evolved tests, etc. On the other hand, the *driver* provides all functionality to start/stop/reset the SUTs, and all the code related to instrument these SUTs with search-based, white-box heuristics (e.g., the *branch distance* (Korel, 1990)).

**Fig. 1:**
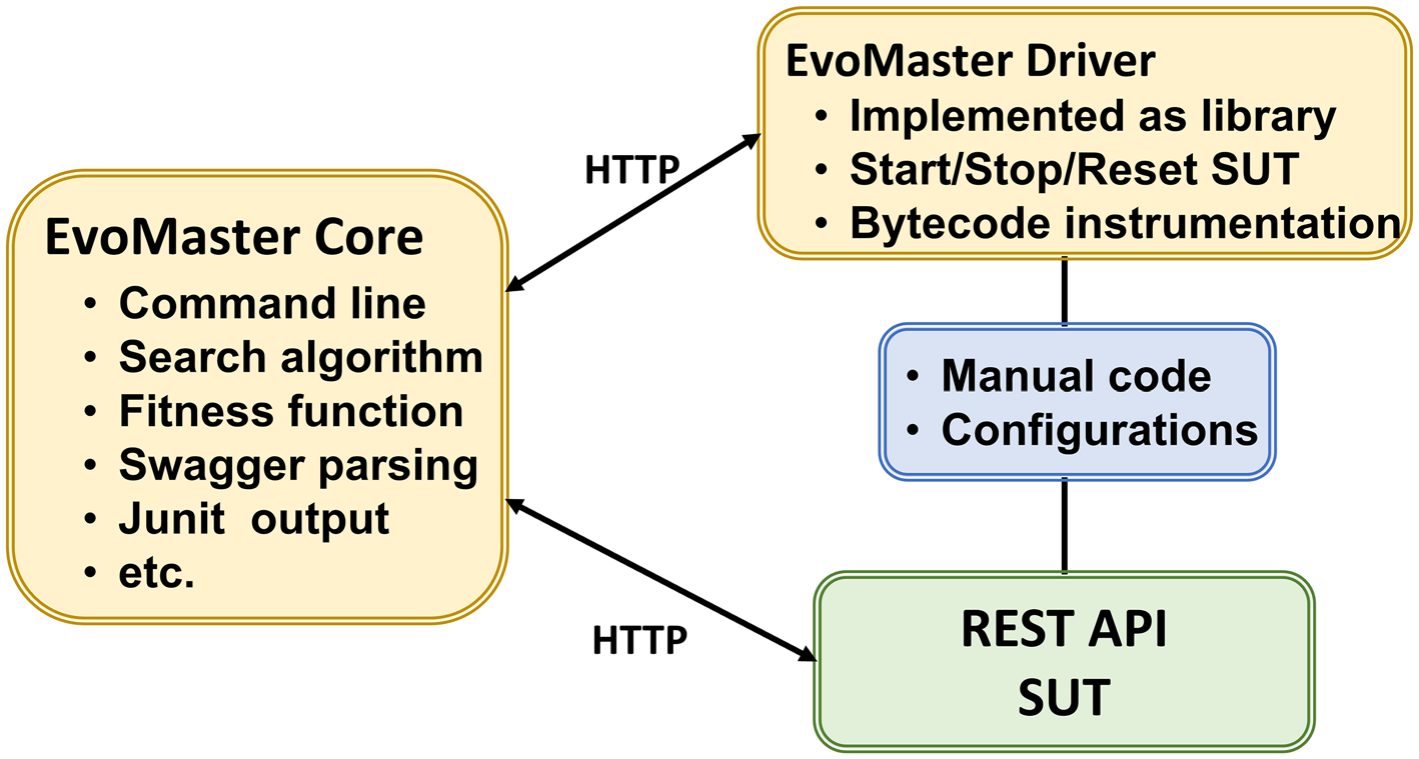
High-level architecture of EvoMaster components (Arcuri, 2018a)

For white-box testing a SUT, the user needs to provide some manual configuration (e.g., how to start the SUT), using the *driver* library. This can then be started as an application, which will open a RESTful API that can be controlled by the *core* (e.g., to collect coverage information after each test case execution, to be able to compute the fitness function). Figure 1 shows a high-level architecture of how *core* and *driver* are related. Note that, with such architecture, supporting another programming environment (e.g., .NET) would just require writing a new driver library, with no needed changes on the core (besides perhaps a new test output format in C#).

**Fig. 2:**
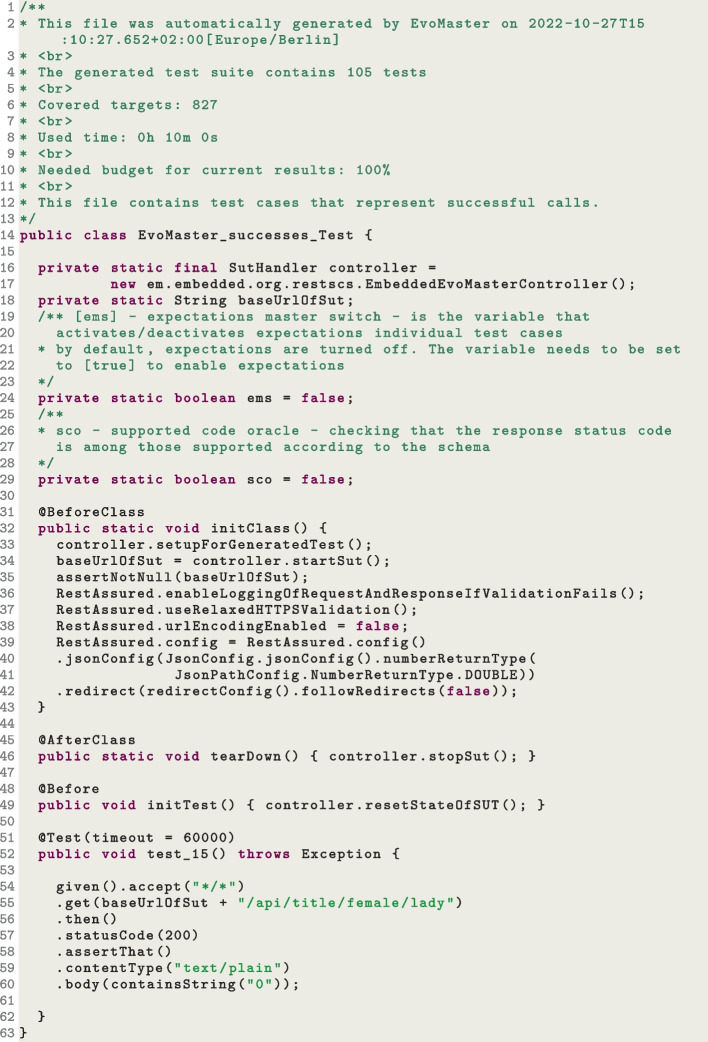
Snippet of JUnit tests generated by EvoMaster when fuzzing a REST API

The output of the *core* are test cases that are “self-contained”, as shown in the example in Fig. 2. These tests will use the driver classes (written by the user, containing the manual configurations), e.g., the configured driver^8^ is instantiated at Line 17 in the example. So the test suite can automatically start the SUT before any test is run (see Line 34), reset the state (e.g., clean SQL database) before each test execution (see Line 49), and then shut-down the SUT once all tests are completed (see Line 46). This is essential to be able to use these tests for regression testing, e.g., to add them to the repository of the SUT, and run on a Continuous Integration system at each new code change. In this example, the test is for fuzzing a JVM REST API (*rest-scs* from EMB (Evomaster benchmark (emb), 2023)), and RestAssured^9^ is employed for performing HTTP requests (see Line 55) and validating responses (see Line 57 for status code and Line 60 for response body).

From an implementation point of view, internally, the *core* makes a heavy use of *object-oriented inheritance*. Most of the code related to the search algorithms and test source code generation are written in a generic way, with specific instances for each problem domain (e.g., REST, GraphQL and RPC). This is done to minimize code duplication, and to ease the adding of new problem domains (e.g., web frontends) in the future.

Another important implementation detail is the use of *dependency injection* frameworks, like for example Guice. In EvoMaster, there is a large amount of data (e.g., hundreds of thousands of evolved test cases) that need to be handled, with data that need to be accessed and used in all different parts of the application, which can span over hundreds of thousands of lines of code. Using a dependency injection framework introduces a non-trivial learning curve for new PhD students and postdocs (as often they might not be familiar with such concept), but it is arguable essential for scaling to large code bases.

On the other hand, for the *driver* library, being a library, architecturally we tried to have it as simple as possible. Considering that it needs to run in the same JVM of the SUT (to do bytecode instrumentation on-the-fly), to avoid classpath library issues, we minimize its number of third-party dependencies. This is the reason why it is written in Java instead of Kotlin (as the use of Kotlin requires importing the libraries of Kotlin as dependency). For the few cases in which we had to use third-party libraries in the driver, we made sure to *shade* them. This means automatically (e.g., with a Maven plugin) modifying their package structure to avoid any class-name clashes. One downside of this technique is that it increases the driver’s JAR size.

For the *core* process, we provide installers for the main operating systems, like for example Windows and Mac. Therefore, we do not have any special restriction on which JDK to use (8, 11 or 17), as we ship it with the installer. This is not the case for the *driver* library, though. The used JDK depends on the SUT, as the same JVM will need to run both the SUT and the code of the driver library. To make the use of EvoMaster as widespread as possible, we currently support the older JDK 8, and make sure the library can still run without problem on all the following, most recent JDK versions.

## Experiment parameters

### Problem definition

When implementing a research prototype, it is common to end up having many *parameters* to configure it. Research prototypes are built to push forward the boundaries of science, answering existing open research questions. When designing a novel algorithm to answer one of such open research questions, there can be several configurations and details that might need fine tuning and experimentation. Before running any experiment, it would not be feasible to know what are the best configurations for a novel algorithm. Therefore, such tools need a way to experiment with different configurations.

The more a research prototype grows, e.g., when used in different studies, the more parameters might need to be added. For research tools developed throughout several years, it is not unheard that they might end up with *hundreds* of tunable parameters. Without a proper engineering handling, dealing with so many parameters can become very time consuming and error prone. As this issue likely applies to most research prototypes (at least in software testing research), it is of paramount importance.

### Parameter handling in EvoMaster

When building a research prototype like EvoMaster, one of the main challenges is how to deal with the parameters used to configure it. This is critical, as how experiments are run in the different empirical studies strongly depends on these parameters.

As discussed in Sect. 1, we need to deal with several parameters in order to customize how EvoMaster is run. For a test case generator tool, perhaps the most important parameter is for how long to run the tool. For randomized algorithms, the longer they are left running, the better results one can expect. However, practitioners are not going to wait forever to obtain results. Some might be willing to wait 24 h, others might want to see results in a few minutes. As such, controlling the duration of the search is an important parameter that we need to enable the user to configure.

At the time of this writing, EvoMaster is a command-line tool, where parameters can be given as input on the command-line. For example, after it has been installed (e.g., on Windows, using its *.msi* installer), EvoMaster can be run with:
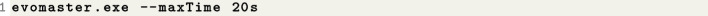


This will run EvoMaster for 20 s, controlled by the parameter maxTime. Depending on the programming language, there are different existing libraries to deal with the handling of console input parameters, like JOpt-Simple^10^ for Java.

**Fig. 3:**
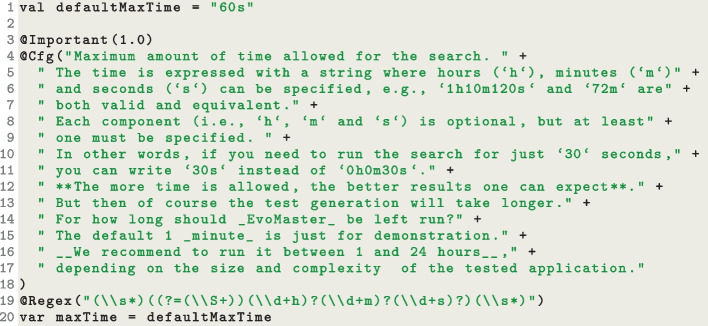
Snippet of the field maxTime in the class EMConfig

In EvoMaster, all parameters are grouped in a single class, called EMConfig. Note: when referring to existing classes/code, we are considering the current version of EvoMaster 1.5.0. But, as EvoMaster is under development, some names might change in the future. Therefore, if there is going to be any mismatch with future versions of EvoMaster, we refer the reader to the long storage of EvoMaster provided by Zenodo, i.e., (Arcuri et al., 2022). EMConfig, which is written in Kotlin, currently contains 161 parameters. Figure 3 shows the code related to the parameter maxTime.

One important aspect here is the use of annotations, like @Important, @Cfg and @Regex. We built custom annotations to enhance and fine-tune how these parameters are handled. All EvoMaster parameters are marked with the annotation @Cfg, which requires us to provide documentation for them. @Important is used for when documentation is generated (discussed later), whereas @Regex is a constraint applied on such parameter.

When EvoMaster is started, and parameters are set, all constraints on those parameters are checked (besides @Regex, there are others like @Min, @Max, @Url, @Folder and @FilePath). For example, calling EvoMaster with something like –maxTime foo will give the following error message:
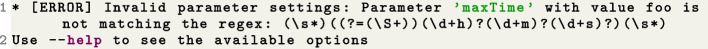


The validation of parameters is essential, especially for parameters that are used at the end of the search. For example, outputFolder (which controls where the generated files are going to be saved) is marked with the constraint @Folder, which checks if the provided string is a valid path for a folder on the current operating system. If you are running EvoMaster for, let us say, 24 h, you want to make sure that a wrong path string is identified immediately, and not crashing/failing the application at the end of the search.

To see all the available options, and their documentation, we can use the option –help. The documentation is based on the strings in the @Cfg annotations, the constraints (if any) and the default values of these parameters. The documentation is then built automatically, via reflection on all the annotations. Furthermore, we also generate documentation in Markdown (implemented in the class ConfigToMarkdown), outputted in a file called options.md. This documentation file is added to the repository of EvoMaster itself, as it then makes it quite simple to access the documentation online^11^. Configurations marked with @Important will show up at the top of the documentation file, whereas the ones marked as @Experimental issue a warning when used. These latter configurations are for settings that are still under development, and/or not fully tested. If for any reason after a change in EMConfig the documentation options.md is not recreated, the build fails, as the consistency of the documentation is checked in the ConfigToMarkdownTest JUnit file.

With the passing of the years, new parameters are added, whereas others might not be any longer relevant. Usually, new added parameters are tuned when carrying out empirical studies to evaluate their effectiveness, and then left as they are afterwards (i.e., no further tuning). So far, we have not faced the issue of deprecating existing parameters, although of course we might face it in the future. However, it might well be that modifying the value of old tuned parameters might have side effects when used with new features. Side effects might include not only performance losses, but also possible faults in EvoMaster that lead to crashes.

We try to make sure that the parameters marked as @Important can be used and modified without any issue. The others are internal parameters, which we do not expect practitioners to modify when they use EvoMaster to fuzz their APIs. Therefore, we do not perform validation techniques like *combinatorial testing* (Nie & Leung, 2011) on these parameters.

It might well be that the best setting for a parameter $X=k$ done in a study might not be optimal years later when a new parameter *Y* is introduced, as *Y* might influence the impact of *X*. When introducing a new parameter *Y* it is not really viable to study its effectiveness compared to all different combinations of all the other existing parameters (e.g., *X*), as those could be hundreds. But choosing new settings for old parameters to take into account the possible impact of new parameters is something that might pay off in terms of performance improvements. We have not carried out such kind of new tuning experiments yet, so we are not currently able to recommend how often and when to do them.

### Lessons learned

Building this kind of infrastructure for handling parameters in this way is not trivial (the reader is encouraged to look at the code of the classes EMConfig and ConfigToMarkdown under the core module, especially if they want to use them as inspiration for their own prototypes). But it is a one-time cost, which then makes the adding and handling of new parameters very simple. As each new scientific study leads to adding one or more parameters, in the long run it pays off significantly (especially when dealing with more than a hundred parameters). Furthermore, it significantly simplifies the analysis of empirical studies, as discussed in the next section.

Among the different implementation efforts that we have done to improve the architecture of EvoMaster, our infrastructure to automatically handle the adding of new parameters is by far among the ones that paid off most throughout the years. Spending time in this kind of coding effort is something we can strongly recommend for new (and existing) research tools.

## Experiment scaffolding

### Problem definition

One of the main goals of building a research prototype is to carry out empirical studies to answer research questions. Considering different parameters to experiment with, and considering the different subjects used as case study (e.g., different software projects for software testing experiments), manually running and analyzing tens, hundreds or even thousands of experiments can be tedious and error prone. Building a scaffolding infrastructure to automatically run and analyze experiments is hence very important. This is particularly the case when using the same tool in different studies throughout the years, as most of the analyses will be similar. If engineered for re-use, most of the experiment scaffolding can be efficiently adapted from study to study with minimal effort, saving consistent amount of time.

### Running experiments with EvoMaster

When running experiments with EvoMaster, we usually need to experiment with different parameters on a series of different systems under test (SUTs). As EvoMaster uses randomized algorithms, experiments need to be repeated with different random seeds (this is controlled with the parameter –seed), typically 30 times per parameter/SUT combination (Arcuri & Briand, 2014). As experiments on system testing can be quite computationally expensive, this often results in very time-consuming empirical analyses, which require clusters of computers to run. For example, if considering 30 repetitions for 10 SUTs, on 2 different parameter settings, this results in $K= 30 \times 10 \times 2 = 600$ experiments to run. If each experiment takes 1 h (not uncommon for system test generation), that would result in 25 days of computation, if run in sequence. As soon as we involve more SUTs and configurations to experiment with, such computational resource requirements will increase significantly.

**Fig. 4:**
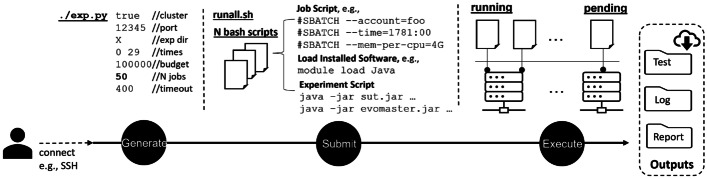
An overview of conducting EvoMaster experiment on the pipeline with exp.py

To simplify the running of all these *K* experiments, we rely on Python scripts to set them up. The idea here is to automatically generate *N* bash scripts, each one containing one or more experiments out of the *K* to run. The reason here is that many research institutes and universities around the world do have access to clusters of computers to run experiments, and those usually are submitted as bash scripts. If a user can run *Z* jobs in parallel, the *K* experiments can be distributed over $N\ge Z$ bash scripts. Figure 4 shows an overview of this pipeline that is used for conducting EvoMaster experiments. As shown in the figure, any user could remotely access the pipeline with Secure Shell (SSH) login. For example, currently all researchers in the Kingdom of Norway have access to high-performance computing resources^12^, where each user can run up to $Z=400$ jobs in parallel. In addition, files could be transferred between a local machine and the pipeline with SSH file transfer Protocol (SFTP) or Secure Copy (SCP). For instance, to conduct EvoMaster experiments, we upload the Python script (e.g., exp.py), an executable jar of EvoMaster (evomaster.jar), and a set of executable jars for SUTs.

#### Generate

The Python script (e.g., exp.py) used to generate the *N* bash scripts needs to take into account several properties. For example, not all SUTs have the same computational cost, and the algorithm used to distribute the *K* experiments over the *N* files takes that into account. It needs to be parametric, as the number of repetitions might vary. Also there are *several* parameters that need to be set that are different from the default values in EvoMaster. For example, when running experiments, we need to make sure that the CSV statistics files are generated (e.g., which include info like code coverage), and use a different stopping criterion (e.g., number of fitness evaluations instead of execution time, to make the experiments easier to replicate).

#### Submit

To process a job with the bash script, the script needs to be specified with info such as time which is an estimated time cost for the job. In addition, we could configure options to employ the computational resources of the pipeline, e.g., mem-per-cpu for memory usage regarding the job. Moreover, the pipeline is equipped with a list of commonly used software that could be used in the experiment, e.g., Java in our experiment (see *Load Installed Software* in Fig. 4). With the script, we also need to specify commands to process the experiment (see *Experiment Script* in Fig. 4) that is automatically handled by the Python script (e.g., start the *driver* for a SUT, then run EvoMaster to generate tests for such SUT). With the pipeline, a job is submitted with sbatch command. To ease the submission process, a bash script named runall.sh is generated by the Python script to submit all generated bash scripts with one command, i.e., ./runall.sh.

#### Execute

After the jobs are submitted, the pipeline would schedule and distribute the jobs into various compute nodes to process on. However, based on its current resource state or the maximum number of jobs (i.e., $Z=400$) which could be executed in parallel per user, a submitted job might be in a state of pending as shown in Fig. 4.

#### Outputs

To collect results of the experiments, we can download the outputs from the pipeline. In the context of SBST, with EvoMaster experiments, we mainly collect three results, i.e., *Test* files for all generated tests, *Log* files for all logs from SUTs and EvoMaster that could be used to debug faults, and *Report* files for all statistics info with CSV format (e.g., configured parameters in this experiment, the number of identified potential faults per run, the number of covered testing targets at 50% of budget used by search).

There are cases in which a researcher might want to run experiments on a local machine (e.g., a laptop) instead of a cluster. This might be the case for when a SUT requires specific software that cannot be installed on the available cluster (for example, we faced this issue when some SUTs required databases like Postgres via Docker^13^). Our scripts can handle these cases as well (e.g., whether the experiments should be run on a cluster or not is a boolean parameter that must be given as input argument).

All this scaffolding for running experiments does not vary much from study to study. The main difference is on what parameters to experiment. So, given the same Python script, it can be simply copied &pasted, with the parameter configuration changed for the current experiments. This approach of using a Python script to generate *N* bash scripts goes back from the time of running experiments with EvoSuite (Fraser & Arcuri, 2011), and it has been refined throughout the years.

**Fig. 5:**
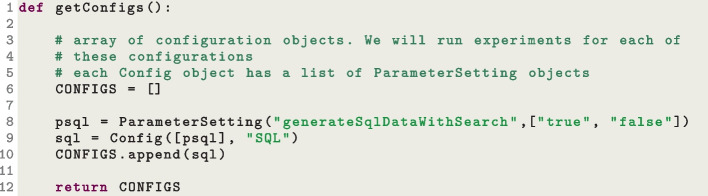
Modification to exp.py to run experiments on the –generateSqlDataWithSearch parameter

The folder scripts in the EvoMaster repository contains the current template exp.py we use when setting up new studies with EvoMaster. For example, let us say we want to replicate one of the analyses related to the handling of SQL data (Arcuri & Galeotti, 2020a). The insertion of data directly into the database is controlled by the parameter –generateSqlDataWithSearch. The snippet in Fig. 5 shows how to modify the script exp.py to run experiments with EvoMaster with 2 different configurations: with and without SQL handling.

Before we can run any experiment, we need to select SUTs as subjects for experimentation. Throughout the years, we collected several SUTs in a single GitHub repository, called EMB (Evomaster benchmark (emb), 2023), and provided scripts to build and install them. The script exp.py refers to the SUTs in EMB (e.g., an environment variable must be set to specify where EMB is installed on the local machine). If one does not want to use all the SUTs in EMB, those can be commented out from the exp.py script. For example, in this paper we are going to use only 4 SUTs (to make these experiments easy to run on a laptop in a not too long amount of time, roughly 10 h in our case).

On a command shell, we can then run:
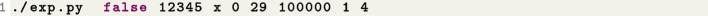


This will generate a folder called “x”, containing 4 bash scripts (one per SUT), each one having 2 experiment settings (with and without SQL handling), repeated 30 times with different seeds, for a total of $4 \times 2 \times 30 = 240$ experiment runs. Each experiment is run up to 100,000 HTTP calls as search budget. Inside such folder, a script called runall.sh is generated, which helps in running all these scripts (not essential when there are only 4, but useful when dealing with hundreds of them that need to be scheduled on a cluster). On a cluster, all the bash scripts will be scheduled with the command sbatch. On the other hand, on a local machine they will be just all started as background processes. As there is no guarantee that *N* jobs would finish at the same time, to better exploit the available resources, we implemented a schedule.py script to schedule them. Given *N* bash scripts, the schedule.py will run *Z* of them in parallel (with $Z \le N$), starting a new bash script as soon as a previous one is completed. *Z* can be chosen based on available resources (CPU cores and memory). More details on these exp.py and schedule.py scripts, and how to use them, can be found in our documentation^14^.

When running experiments in which it is important to keep precise track of used resources (e.g., memory and CPU), there are frameworks that help in carrying out and analyzing experiments, like for example BenchExec (Beyer et al., 2019). It would not be directly applicable for the type of experiments we run with EvoMaster (e.g., due to requirements like “ [tool] *does not require external network communication during the execution*” (Beyer et al., 2019)), but it is something to consider when dealing with other kinds of testing problems (e.g., testing of CPU-bound applications written in C that run on Linux).

### Analyzing the results

**Table 3:** Experiment results on 5 SUTs from EMB, where the impact of –generateSqlDataWithSearch on target coverage is analyzed

SUT	Base	SQL	$\hat{A}_{12}$	p-value
*catwatch*	1198.4	1279.4	0.97	<0.001
*features-service*	715.8	721.6	0.71	0.005
*rest-news*	334.8	334.5	0.50	0.970
*rest-scs*	838.9	856.0	0.62	0.107

Once the experiments are started with runall.sh (or schedule.py), all generated tests will be inside the folder x. Furthermore, CSV statistics files are also generated (by using the parameter –statisticsFile). Note that such files could also be accessed with the experiment on the pipeline as shown in Fig. 4. Those statistics files will contain two types of information: *static* information about the configuration used in the experiments (i.e., all the parameter settings), and *dynamic* information on the results of the search (e.g., different code coverage metrics). This data can then be used to analyze how successful a new technique is. For example, let us say we want to study the impact of –generateSqlDataWithSearch on achieved target coverage (EvoMaster optimizes for several different metrics, like line and branch coverage). We can study the achieved coverage on the *Base* version (i.e., when that parameter is set to False) compared to the new improved *SQL* version (i.e., True). To properly analyze such comparison (Arcuri & Briand, 2014), we need to use the appropriate statistical tests, like for example the Wilcoxon-Mann-Whitney U-test, and standarized Vargha-Delaney $\hat{A}_{12}$ effect-size. Table 3 shows the results of these experiments.

In these experiments, we can see that the SQL handling provides statistically significant improvement for 2 APIs (i.e., *catwatch* and *features-service*), but not the others. The lack of statistical significant results on *rest-scs* is expected, as that SUT does **NOT** use a database. So, in theory (unless there is some software fault in EvoMaster) the ability of adding data directly into a database should have no effect here. However, results seem better for the *SQL* configuration, with higher average number of covered targets and effect-size $\hat{A}_{12}$. However, the *p*-value of the U-Test tells us that there is not enough evidence to claim the two configurations give different results at $\alpha =0.05$ confidence level. This is an important reminder of the stochastic nature of this kind of algorithms, and the fact that even with 30 runs there can be quite a bit of variability (Arcuri & Briand, 2014). Using statistical analyses is essential to avoid drawing misleading conclusions. The case of *rest-news* is quite interesting as well. Here, there is no improvement in the results, with very high *p*-value. However, in our original analysis in Arcuri and Galeotti (2020a) there was improvement for this SUT. Being able to add data directly into the database might not be so important if the testing tool is able to effectively use the endpoints of the API itself to create the needed data. As EvoMaster has been improved since we carried out the analyses in Arcuri and Galeotti (2020a), the introduced novel techniques like *resource handling* (Zhang et al., 2021) and *adaptive hypermutation* (Zhang & Arcuri, 2021a) might have been enough for this specific SUT.

**Fig. 6:**
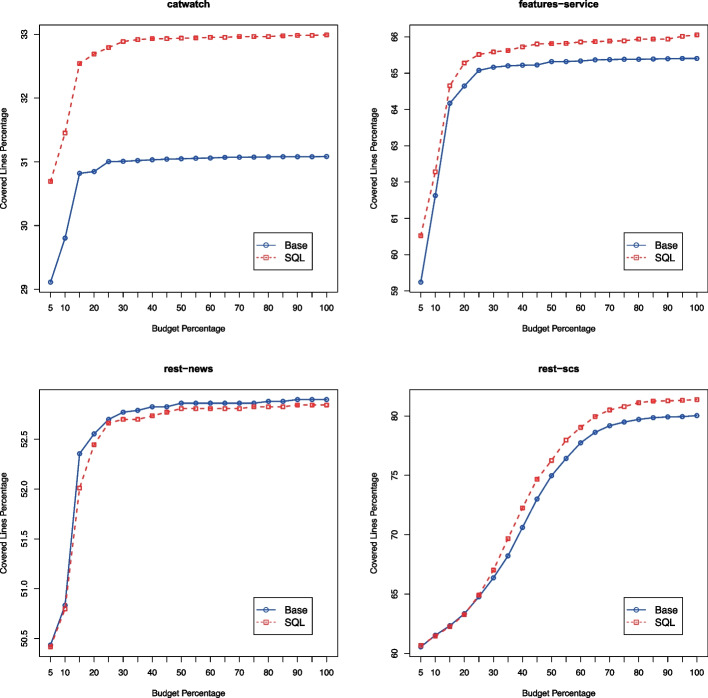
Average (out of 30 runs) number of covered targets throughout the search, for the 2 configurations *Base* and *SQL*

Choosing the right search budget for carrying out experiments is not trivial. We usually employ a budget of 100,000 HTTP calls, as, for our SUTs used in our case studies, those take roughly between 20 and 60 min (depending of course on the hardware used). Ideally, search budgets should reflect the actual usage of practitioners in industry. This could be minutes, hours or days, depending on the testing scenarios and hardware availability. Given enough time, even a naive random search (as well as a brute force enumeration of all possible test cases) can in theory achieve full coverage (Arcuri et al., 2012), albeit in a non-viable amount of time. Therefore, it is interesting as well to study the performance of algorithms at different points in time. To achieve this, in EvoMaster we have the option (using –snapshotStatisticsFile) to output a snapshot of the current results at different points in time (controlled by the parameter –snapshotInterval). These generated snapshot CSV files can then be used to create performance plots like the ones in Fig. 6.

### Dealing with failures

When running this type of experiment which strongly relies on resources from the operating system (e.g., TCP ports), and that can run for hours or days, it is important to understand that some experiments might fail, sporadically (especially if too many experiments are run in parallel on the same machine). Also, there might be cases of faults in the tool which only manifest on specific SUTs. As each experiment run generates one CSV file, it is important to check (e.g., with a script) at the end of the experiments that the total number of generated CSV files matches the number of experiment runs. In case of issues, to help fixing those, making sure that all the outputs (i.e., logs) of the tool and SUTs are saved to disk is paramount. This can be automatically handled (e.g., by redirecting the standard outputs of these processes to distinct files on disk). When repeating experiments for 30 times (or more) to handle their randomness, missing 1 or 2 runs (e.g., due to the operating system not recycling TCP ephemeral ports fast enough) is usually not a big issue, as long as the results of the statistical tests are still significant (e.g., at $\alpha =0.05$ threshold for the *p*-values). However, if many runs are missing, those require investigation, and re-run all the experiments once the issues are fixed.

Fixing bugs and re-running experiments is viable when one is the author of the used tool. However, when comparing tools from different authors (e.g., Zhang and Arcuri (2022)), that is usually not the case. Furthermore, the SUT itself could crash due to faults. This is usually good for practitioners (as fuzzers are used to find if there is any fault), but it can complicate the analysis of the experiments (e.g., when trying to collect code coverage results). For the specific case of Web APIs though, this is usually not a problem. Web APIs are usually run inside HTTP servers (e.g., Tomcat). If there is a fault in the API which leads to an uncaught exception, then the HTTP server simply catches it and returns an HTTP response with status code 500. The whole SUT would “crash” only if there is a fault in the code of the HTTP server, which is an occurrence that we have not experienced yet.

### Automation of the analyses

Using this kind of script like exp.py to set up the experiments also helps with *replicated* analyses from third-party research groups. In addition, this setup helps when preparing *replication packages* for academic Software Engineering conferences. We will go back on this point later in Sect. 9. As part of the Git repository of EvoMaster itself, we store not only the PDFs of all the published articles based on it, but also all the exp.py files used to run the experiments^15^.

One important aspect to further discuss is how a table such as Table 3 is created. Of course, this can be done manually, but it is error prone (i.e., calculate all the statistics with a tool, and then fill the table manually). Furthermore, it is tedious and time consuming, especially when experiments need to be repeated (due to some mistakes), or many tables need to be filled/updated at the last moment just before an academic conference deadline. It would be better to *generate* such tables automatically, including all their statistical analyses. This is possible, at least when using text editing tools like Latex. The idea is to write the analyses in a scripting language (we use R, but Python would do as well), and output as a text a table in Latex format, which can then be imported in the article with the Latex command input. Such R script will read the CSV files of the experiment results to do such analysis.

**Fig. 7:**
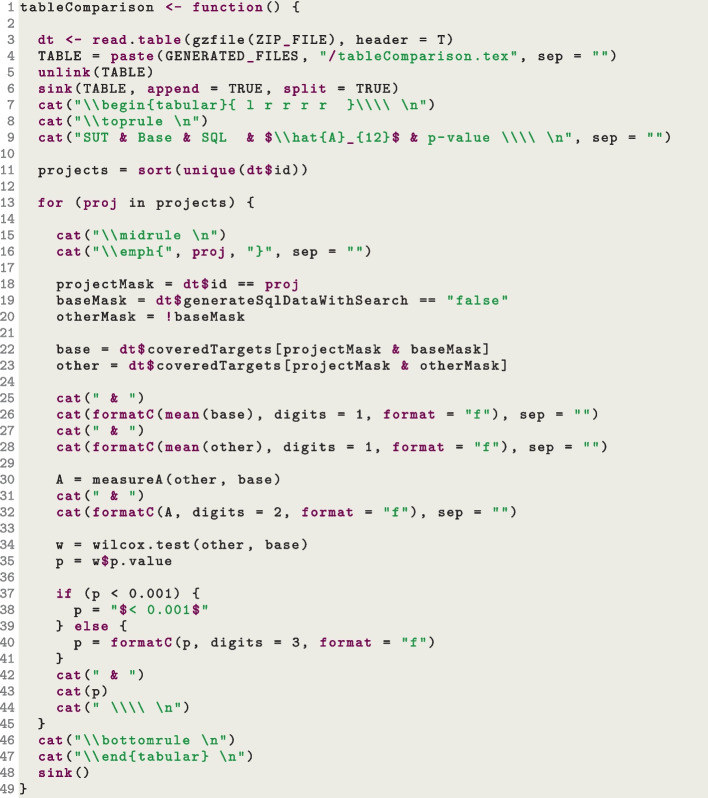
Snippet of R code used to generate Table 3

Figure 7 shows a snippet code of the R function used to generate Table 3. It outputs a text file called tableComparison.tex (Line 4). In this script, the data is first loaded (variable dt, Line 3), and then grouped by SUT (called proj in the script, Line 11). Then, for each SUT (Line 13), the data is divided based on the parameter generateSqlDataWithSearch (which is a column in the CSV file, Line 19), and the two sets of data base (Line 22) and other (Line 23) are compared (Lines 30 and 34). The Latex commands used to create the table are outputted as text with the R command cat (e.g., Line 26), where the standard output is redirected to the tableComparison.tex file (R command sink, Line 6). This table can then be easily included in a paper with the following Latex instructions:
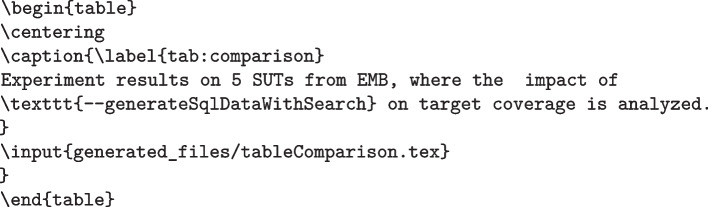


The plots in Fig. 6 have been generated automatically with the same approach, using R commands like pdf() and plot() to generate 4 different PDF files, and then imported in Latex with \includegraphics.

### Lessons learned

What is discussed in this section might sound convoluted, but, again, it saves a huge amount of time in the long run. Especially considering that most scientific articles will have similar types of analyses, and very little effort is needed to adapt these scripts from article to article. This is because each parameter setting (e.g., –generateSqlDataWithSearch) is just yet another column in the generated CSV files, where data can be filtered on (e.g., as done at Line 19 in Fig. 7).

Setting up a whole automated pipeline to run the experiments and analyze them is not trivial, and requires effort and expertise. However, we can highly recommend it for tool prototypes that are going to be used in more than just a couple of studies. For example, today when we run new experiments with EvoMaster on some novel techniques we have designed, with the experiment scaffolding discussed in this section it is just a matter of few minutes to set up all the experiments and their analyses.

## E2E tests for EvoMaster

### Problem definition

Software research prototypes are still software, and, as such, they can have software faults. Software faults can lead to errors and publishing completely invalid results. As for any software, to avoid this issue research prototypes need to be tested.

Most of the software testing practices would apply to research prototypes as well. However, research prototypes do also have specific challenges when dealing with their testing, which are worth to discuss in more detail.

### Test cases in EvoMaster

**Fig. 8:**
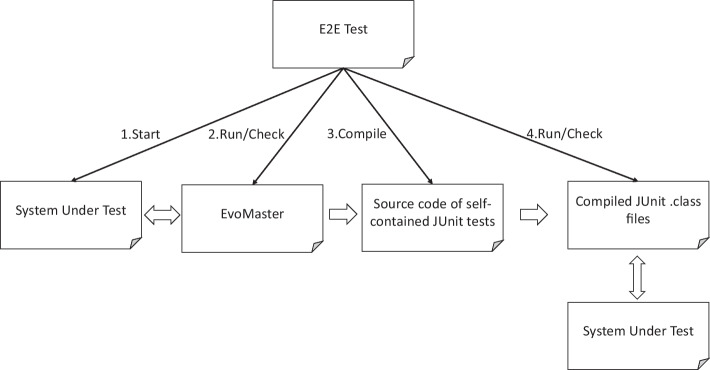
Overview of EvoMaster E2E tests

As any piece of software, EvoMaster itself needs to be tested. Because it is not an enterprise application, but rather a tool, EvoMaster cannot be used to test itself. So, we rely on manually written tests.

The writing of test cases for EvoMaster is similar to any other type of software, i.e., there is typical distribution of unit tests, integration tests and system tests. General guidelines on software testing practices would apply here as well. However, one peculiarity that is worth to discuss in more detail is how we write End-to-End (E2E) tests.

An E2E test in EvoMaster would require to run the tool on a SUT, generate test cases, compile them, run them, and then verify if any specific level of code coverage and fault detection has been achieved. Figure 8 depicts such steps. In EvoMaster, we do all of this, automatically. The reader is encouraged to look at the (currently) 88 test suite files (with name extension EMTest, written in JUnit) in the e2e-tests folder. From a JUnit test case *T*, we programmatically start the SUT, we programmatically run EvoMaster on it, outputting the generated test cases *Z* to a specific folder. Once EvoMaster ’s search is completed, we verify (with JUnit assertions) different properties (e.g., typically if specific SUT endpoints have been called and they returned some expected HTTP status code). Then, from *T* itself, we first call the compiler to compile the source code of *Z*, and dynamically load such compiled tests *Z* and run them programmatically as well inside *T* (using JUnit’s APIs directly). Note, if any of these steps fails (e.g., EvoMaster has a fault and its generated tests are not syntactically correct, so the compiler would fail), then the E2E test *T* fails. However, when doing all this, there are a few challenges.

First, we need SUTs. We simply implemented them, and added them to the repository of EvoMaster (under such e2e-tests folder). The goal here with these tests is to make sure that EvoMaster can work on different kinds of technologies used in the SUTs (e.g., frameworks like Spring, different versions of OpenAPI schemas and databases like Postgres and MySQL). However, the SUTs cannot be too complex, i.e., we cannot let EvoMaster run too long on those SUTs, as otherwise the build will take an unreasonable amount of time. All these E2E tests are run automatically as part of the build, by using Maven. The current build takes around 1–2 h due to these E2E tests^16^. So, on each of these SUTs, we run EvoMaster for less than a minute. On each E2E test we verify a specific feature *X* of EvoMaster. The idea is that, without *X*, it should not be possible to fully test the SUT. However, when *X* is used (and its implementation is not faulty), then the SUT becomes trivial to test (and so EvoMaster would not need to be run long on such an SUT).

**Fig. 9:**
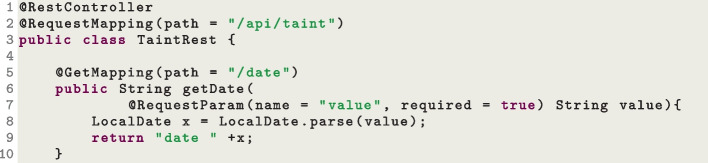
Snippet code of the class TaintRest, showing implementation of a GET HTTP endpoint, using the Spring framework

Figure 9 shows parts of a REST controller of one of the SUTs we use for the E2E tests. That specific GET endpoint expects a string parameter as input. However, internally, a call to LocalDate.parse is done (Line 8), i.e., the input string is checked to see if it is a valid date. As the schema has no information on such constraint on this value, it is extremely unlikely that a random string would pass such constraint. When given a non-valid date as input, then LocalDate.parse will throw an exception, and the framework (Spring in this case) will just return an HTTP response with status code 500 (*Internal Server Error*). However, thanks to white-box heuristics like *Testability Transformations* and *Taint Analysis*, this code is trivial to cover for EvoMaster  (Arcuri & Galeotti, 2021a). The E2E test TaintEMTest is then checking that it is possible for EvoMaster to craft a valid HTTP request that returns a 200 HTTP status code on this endpoint.

### Dealing with flakiness

EvoMaster uses the evolutionary algorithm MIO (Arcuri, 2018c) enhanced with adaptive hyper-mutation (Zhang & Arcuri, 2021a) to evolve test cases. As it is a *randomized* algorithm, when running a search there is no guarantee that an optimal solution is found. Running the algorithm twice on the same inputs can lead to very different results. This not only brings challenges on how to evaluate the effectiveness of these algorithms (which requires repeated experiments and use of statistical analysis (Arcuri & Briand, 2014)), but also on their testing. For example, even if the SUTs that we develop for the E2E are simple, there is still a non-zero chance that a test can fail, due to the randomness of EvoMaster. *Flaky* tests bring a lot of problems, especially for debugging.

One solution to address this issue is to have a full control on the sources of non-determinism in the algorithm. Given a pseudo-random generator (e.g., the Java API java.util.Random) needed by the search algorithm, we use only one single instance inside EvoMaster, initialized with a specific seed. When EvoMaster is used by practitioners, the seed is taken from the CPU clock. On the other hand, when we run E2E tests, the seed is fixed (e.g., to 0), using the parameter –seed. Even if a test fails, if we run a second time (e.g., for debugging), we should get the same results and execution trace.

Unfortunately, though, pseudo-random generators are not the only source of non-determinism. For example, iterating over un-ordered data structures like *Set* might result in a source of non-determinism (i.e., the order in which elements are traversed can be each time different), depending on the actual implementation of such data structures (this is a concrete problem for some specific implementations in the Java APIs). Care is needed to make sure to only use deterministic data structure implementations (e.g., HashSet vs. LinkedHashSet). Another major source of non-determinism is the idempotency of some HTTP calls (e.g., depending on the Operating System, at times some calls can be automatically repeated, possibly leading to slightly different results, especially related to logs).

**Fig. 10:**
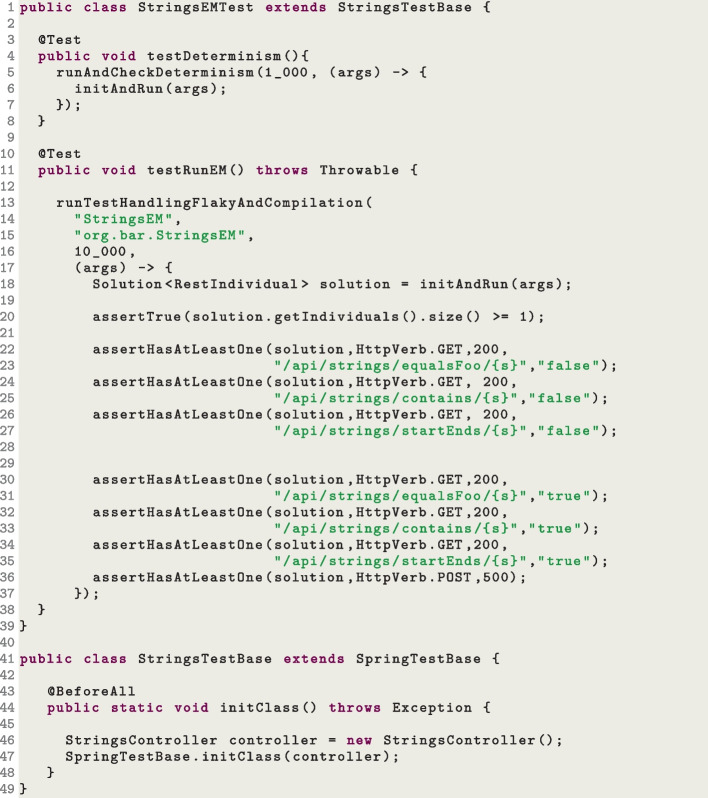
Snippet code of the test suite class StringsEMTest

To make sure the different sources of non-determinism are properly taken care of (or at least their effects are negligible), we have specific test cases to check this. The idea is rather straightforward: when running an E2E test, we store all of its logs (and we make sure to activate *verbose* logging); then, we run the same test twice, and we verify that the logs are *exactly* the same. When we do this, in the logging configurations we need to make sure to avoid any time information (e.g., timestamps of logs), otherwise the tests will always fail. This is done with the option –avoidNonDeterministicLogs true. Furthermore, for these tests we need to disable all the time-related features of the search algorithm (e.g., current extension of MIO is using the execution time of each endpoint to prioritize them), which is done with the option –useTimeInFeedbackSampling false. The test StringsEMTest.testDeterminism is an example of this kind of tests (see code in Fig. 10, Line 4).

Although the SUTs we write for the E2E tests are simple, there is still a possibility (even if very low) that, for some seeds, a test case would fail. Dealing with this kind of flaky tests by simply re-run the test cases up to *N* times (e.g., $N=3$) would not work, as we are controlling the source of non-determinism (as previously discussed). The solution here is that, we still re-run the test, but, this time, with a different seed (specified with the –seed option).

All these logic discussed so far in this section (e.g., compilation and flakiness handling) are shared among practically most of the E2E tests. To avoid repetition, we abstract most of this logic in a library, which then lead these test cases to be rather short and easy to write and maintain. Figure 10 shows the implementation of the test suite class StringsEMTest. The first test testDeterminism() (Line 4) is running EvoMaster on the SUT defined in the controller StringsController (Line 46). EvoMaster is run twice for 1000 fitness evaluations, and the logs are then compared. All this logic is done in the function runAndCheckDeterminism() (Line 5), which can be re-used by all these kinds of tests. On the other hand, the test testRunEM() (Line 11) is running EvoMaster for 10,000 fitness evaluations, in which we then verify at the end of the search that different endpoints are covered with different status codes (as well as checking if some specific values do appear in the payloads of the HTTP responses, Lines 23 to 36). Most of the logic is handled inside the runTestHandlingFlakyAndCompilation() function (Line 13), which is common for all these kinds of E2E tests.

### Lessons learned

As software engineers, and not just academics, we strongly believe in the usefulness of software testing. If it was not the case, we would not dedicate our careers on this research topic. Therefore, as we believe software testing is useful and pays off in the long run, our research prototypes are thoroughly tested. In different words, we follow the saying *Do you practice what you preach?*

Throughout the years, *hundreds of faults* were automatically found in EvoMaster by running our regression test suites. Given the version control system Git, a typical scenario is to develop new features in a *branch*. Only if all test cases pass such features can be merged into the *master* branch by making a Pull Request (PR). If some test cases fail, then the faults should be fixed before the PR can be merged.

Among the different kinds of tests, our E2E tests were the most effective at finding regression faults. Given a large and complex software project, it is not unexpected that apparently harmless changes might have serious side effects on other parts of the code unrelated to the new feature under development. But, as discussed, writing E2E tests for software testing tools is complex, as for example it requires compiling and running the generated tests on-the-fly. With the right engineering effort, such cost can be drastically reduced when writing new E2E tests, although there is still a high one-time cost to build all the needed scaffolding. Still, we strongly recommend to do this, due to the huge savings it gave us throughout the years.

## Technical details

### Problem definition

When dealing with the system testing of web/enterprise applications, there are some specific technical challenges that need to be addressed. In this section, we discuss the three main ones we faced in the development of EvoMaster, namely: *databases*, *authentication* and *multi-threading*.

These selected challenges represent critical features that must be implemented when building a white-box fuzzer for this kind of application. They are not trivial, as there are several edge cases that must be handled. We describe them here in more detail, with some solutions, to enable the readers a useful head-start when working on this kind of systems. The alternative would be to be bound to spend significant time in rediscovering them.

### Dealing with databases

Most of the time, web/enterprise applications interact with databases. The most common databases are SQL ones such as Postgres and MySQL (which we support in EvoMaster), as well as NoSQL ones such as MongoDB (currently not supported). A database will store the status of the application, and it has a major impact on the testing of the SUT. One crucial aspect here is that test cases must be *independent*, i.e., the execution of a test case should not depend on the state modified by previous tests. Otherwise, test outcomes would depend on their execution order, which significantly complicates tasks like debugging. A solution here is that the “state” of the application has to be reset before/after each test case execution.

Simply restarting a database would address this issue at each test execution, but it would be way too naive. Restarting a database takes a non-trivial amount of time, which would end up to be a major performance bottleneck. So, we “simply” clean (i.e., delete) the data in the database at each test execution by executing custom SQL commands. Interestingly, there is no standard approach for “cleaning” a database (as this is a functionality mainly needed for testing), and each database requires slightly different commands. For example, where Postgres allows a single command with a series of table names to TRUNCATE, H2 and MySQL need one TRUNCATE command per table. But, removing data from a table can break the constraints for foreign keys, resulting in the command to fail (and so the data does not get deleted). So, we first need to disable all the constraint checks on the referential integrity (and each database requires a different command for this), then truncate each table one at a time, and finally re-enable the constraint checks. Besides this, all sequence generators (e.g., used for auto-increment columns) need to be reset. And, again, each database requires a different SQL command to achieve this.

**Fig. 11:**
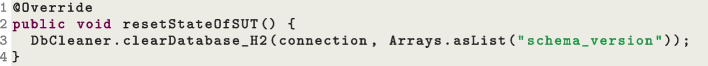
Snippet code of a *driver* class, in which at each test execution a H2 database is cleared

To help with this needed functionality, we developed a library (released on Maven Central) in which the class DbCleaner can be used to reset the state of the database (if any is used by the SUT). The snippet in Fig. 11 shows an example of the implementation the resetStateOfSUT() method in a driver class (needed to be implemented by the user when they want to do white-box testing of their SUTs with EvoMaster). Here, the function DbCleaner.clearDatabase_H2 is used to tell the driver how to reset the state of SUT, which is stored in a H2 database (we provide equivalent functions for Postgres and MySQL as well). This function takes two inputs: a required java.sql connection to the database, and an optional list of table names to skip (i.e., their data should not be cleared). This is needed when dealing with database migration tools such as Flyway and Liquibase (as those use some tables to keep track how the schema of the database itself should be updated when the SUT starts). As resetting the state of a database is an essential feature for most system test generation tools, this DbCleaner utility could be useful as well outside of EvoMaster.

**Fig. 12:**
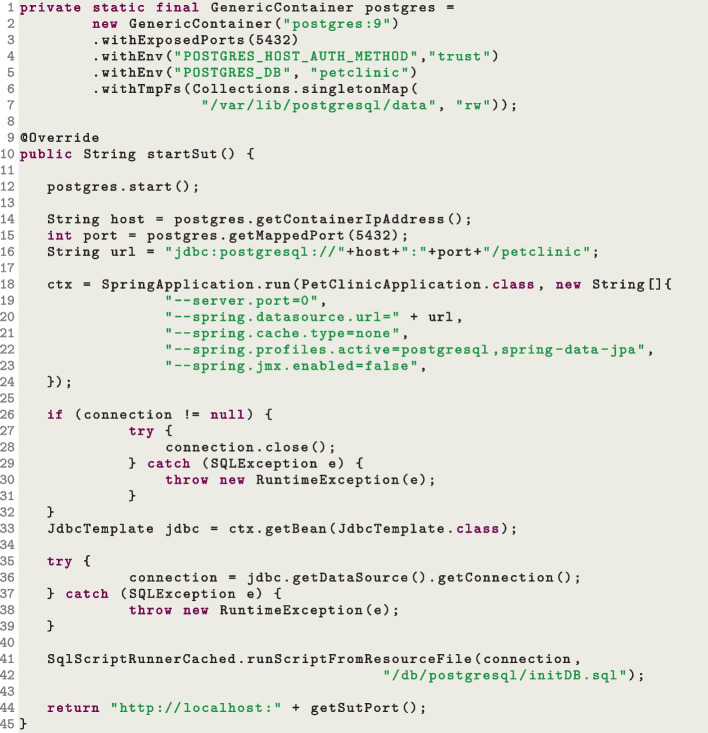
Snippet code of a *driver* class, where a Postgres database is started together with the SUT

For debugging and regression testing, it would be advisable to have test cases that are “self-contained”, i.e., being able to start the SUT and all of its required dependencies (e.g., databases) automatically from the tests themselves. Where starting an application programmatically is rather straightforward with professional frameworks (e.g., Spring), for databases a tester can use embedded ones (e.g., run in the same JVM of the test), such as H2. Where this is not possible (e.g., the SUT uses special custom database features which are not available in an embedded database), nowadays it is rather straightforward to start the actual databases such as Postgres directly from the test cases. This can be easily done using Docker and libraries such as TestContainers^17^. Then, in the EvoMaster drivers these databases can be started before the SUT is started. Figure 12 shows another snippet from a SUT driver, where a Postgres database is started via Docker (Line 12, configured at Lines 1 to 7), and then the SUT (a Spring application) is started programmatically (Line 18), using as input an updated URL for the database connection (Line 16). Note that the generated tests by EvoMaster will then use such startSut() method (Line 10) to start the SUT and all of its dependencies.

For test case generation, it can be useful to analyze all the interactions of the SUT with its databases (if any). In previous work (Arcuri & Galeotti, 2020a), we have extended EvoMaster to be able to intercept and analyze all SQL commands, and use such information to generate better (i.e., higher coverage) test data. At that time, to achieve this, we used the library called P6Spy. However, that requires the user to add such library to their SUTs, and manually modify the database URL connections to use P6Spy (which can be seen as wrapper for JDBC connections). Then, each time a SQL command is executed it would be logged into the standard output, and EvoMaster then reads it.

This approach works (Arcuri & Galeotti, 2020a), but it is a bit cumbersome (especially for the users that need to manually set up P6Spy). Once we have had in place the code scaffolding for *Testability Transformations* (Arcuri & Galeotti, 2021b), we removed the dependency to P6Spy. We rather intercept at bytecode loading all the usages of SQL database interactions, i.e., the use of classes such as java.sql.Statement and java.sql.PreparedStatement, by providing *method replacements* (Arcuri & Galeotti, 2020a) (e.g., see the classes StatementClassReplacement and PreparedStatementClassReplacement). In this way, we can achieve the same results as in Arcuri and Galeotti (2020a) without the need of any manual configuration (it is all automated), plus we get further benefits like being able to track the execution time of each SQL command (e.g., this was requested by one of our industrial partners that use EvoMaster).

One challenge here is in the dealing of PreparedStatement, which is used for interpolated SQL commands (needed to avoid SQL Injection attacks). The interfaces of JDBC do not provide any functionality to retrieve/compute the actual SQL command (e.g., as a string) of a prepared statement (which we need for the analyses in EvoMaster). Databases like Postgres and MySQL provide some unofficial way to retrieve the actual, interpolated SQL commands (e.g., via the toString() method), whereas H2 does not. In this latter case, we had to write the code to do the interpolation ourselves (see the code of the class PreparedStatementClassReplacement).

One issue here is that adding support for a new database is not necessarily simple: there is the need to provide a cleaning utility (e.g., as an extension of DbCleaner), as well as to provide a way to interpolate prepared statements (e.g., needed for PreparedStatementClassReplacement). In EvoMaster, the choice of supporting H2, Postgres and MySQL has been based simply on convenience, i.e., we support the databases of the open-source SUTs we could find for experiments (e.g., H2 and Postgres), as well as the ones from our industrial partners (e.g., MySQL).

### Dealing with authentication

Apart from APIs that are read-only with publicly available data, it is common that web/enterprise applications require some form of *authentication*. This is especially the case for CRUD applications, and also for read-only APIs that deal with sensitive information (e.g., banks and medical records). Typically, authentication is based on logins with ids and passwords, where the obtained authentication tokens are (usually) sent via HTTP headers.

There are several different ways to do authentication, and we do support a few in EvoMaster. Two interesting technical challenges are worth discussing in bit more detail: *hashed passwords* and *dynamic tokens*.

When a user account is created, information on user ID and password are typically stored in a database. However, for security reasons, passwords are not stored in plain text, and are typically *hashed* (e.g., using functions like BCrypt). For example, in the European Union, it is actually illegal to store passwords in plain text, due to General Data Protection Regulation (GDPR)^18^. Hash functions are designed on purpose to be extremely hard to reverse, i.e., retrieve the original password from its hashed value.

**Fig. 13:**
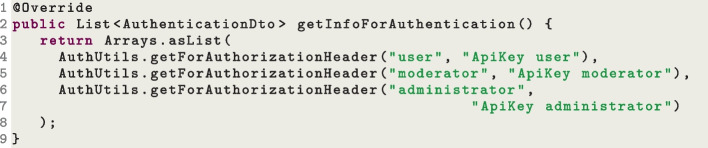
Snippet code of a *driver* class, where the authentication for three users is set up

**Fig. 14:**
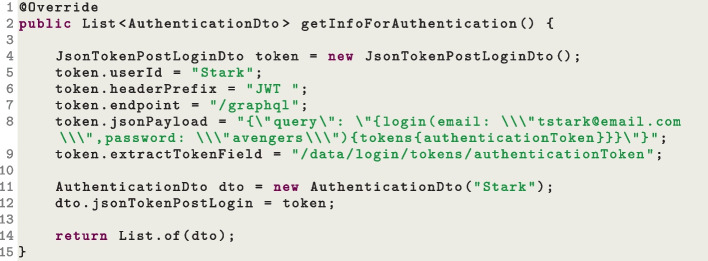
Snippet code of a *driver* class, where the authentication for a single user is set up via JWT

This has an impact on automated test generation. If an API does not have a way to create new users (or create users with specific roles such as administrators), it is not viable to inject valid hashed values into the database (recall that, besides generating sequence of HTTP calls, EvoMaster can also inject data directly into the SQL databases as part of the search (Arcuri & Galeotti, 2020a)). Doing so would require to analyze exactly which hashing algorithm is used in the SUT, and enhance the search in EvoMaster to use exactly the same hashing function (technically possible, but extremely challenging to implement, as the SUT could use any type of hashing function). This is the reason why, for the time being, the setting up of valid user profiles is not automated, but rather left to the user to manually set up in the EvoMaster drivers for the SUTs. To help deal with different kinds of authentication mechanisms, we have implemented a library to support users. Figures 13 and 14 show two different implementations of the driver method getInfoForAuthentication() that we needed for some of our SUTs used in our experiments. During the search, EvoMaster queries this method, and then decides if using any authentication information for the test cases.

The example in Fig. 13 is the simplest of the two, where three users are defined with *static* authentication tokens. Those tokens are simply added directly to the *Authorization* header on each HTTP request. On the other hand, Fig. 14 shows a more complex case in which, for authentication, there is the need to make a POST HTTP request on a specific endpoint, with a specific body payload (including id and password). Then, it needs to extract a JWT token from the JSON body response, and use such token for authentication in the *Authorization* header in all following HTTP messages. In this case, the JWT is *dynamic*, as each new login would generate a different token. This means that each time we evaluate a test case, we need to make such POST login, and add the received token in all following HTTP calls in such test. This is done not only during the search, but also in the outputted JUnit test files.

**Fig. 15:**
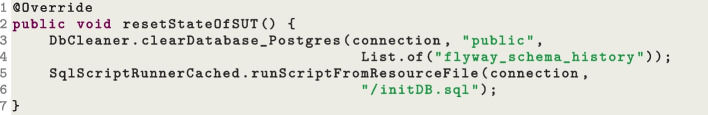
Snippet code of a *driver* class, where the authentication information is added into the database after each reset

Regarding the storing of user info, including hashed passwords, this needs to be manually set up, e.g., in a SQL script, and called each time the state of the SUT is reset. Figure 15 shows another example of a resetStateOfSUT() implementation where, after each database is reset, the SQL script initDB.sql is executed to add user info (including hashed passwords) for the profiles defined in the getInfoForAuthentication() method.

### Dealing with code instrumentation and multi-threading

**Fig. 16:**
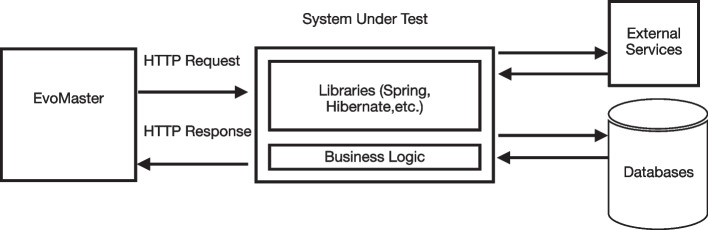
A graphical overview of the computation cost of a test case evaluation

When testing an application, we need to instrument its business logic, to be able to compute its code coverage and different kinds of SBST heuristics (Arcuri, 2019). However, there is no need to collect coverage information of third-party libraries, as those are not what users are interested to verify. For example, for a web service, there is no need to try to maximize code coverage of its HTTP server (e.g., Tomcat), nor of the libraries used to access the database (e.g., Hibernate), nor the code of frameworks like Spring. It is not uncommon that the actual business logic of the SUT is just a very small part compared to all the needed third-party libraries used in the application. Furthermore, for typical *I/O-bound* web applications, the computational overhead of the business logic of the SUT can be small compared to the overhead of sending messages over HTTP, and to all the needed I/O interactions with databases and external services. Figure 16 gives a graphical overview of the computation cost of a test case evaluation. This means that, in most cases, the computational overhead of code instrumentation is practically negligible when we evaluate a test case.

Unfortunately, a case for which this is not really true is for *CPU-bound* applications. An example of this is the SUT LanguageTool, which does different kinds of complex text analyses. Here, the first time we tried EvoMaster on it, our code instrumentation was a major bottleneck, both in terms of computational time (going from milliseconds to *minutes*) and memory consumption. Fixing this required the use of profilers (e.g., YourKit) to point out the major bottlenecks in the instrumentation. Care needed to be taken to optimize the code, using different techniques such as for example *memoized* functions. Drastic performance improvements were achieved, although more still needs to be done (especially in the analysis of regular expressions). Note that these low-level code optimizations were necessary for our code instrumentation, and not the *core* of EvoMaster itself (e.g., the code of search algorithms). This is because the evaluation of the fitness function (i.e., running a test case by making HTTP calls and then retrieving all the metrics collected via the code instrumentation) takes the vast majority of the search time (at times even more than $99\%$). Any code optimization there will have practically little to no effect on performance.

Given *S* the computational cost of the search algorithm (e.g., sampling an individual or mutating an existing one), *H* the cost of making an HTTP toward the SUT and the get its response, and *I* be the cost overhead of the code instrumentation in the SUT, then the cost of evaluating a new individual (i.e., an evolved test case) would be $C=S+H+I$. For most cases, *S* and *I* are tiny compared to *H*. When *I* is high, one thing that could happen is that the HTTP call could *timeout* (note this could also happen regardless of *I* if *H* is high, e.g., due to a bug or simply very inefficient code for some specific inputs). For example, when making an HTTP call, one needs to specify for how long the TCP connection is going to wait (e.g., *T* seconds) before getting a response. If the response does not arrive in time (i.e., $H+I > T$), then the TCP connection does timeout, and the HTTP call fails. Choosing the right value for *T* is not straightforward. Although a high value would prevent most timeouts, it would also mean that some fitness evaluations could become very time consuming. And this could hamper the search because, in the same amount of time, less fitness evaluations would be executed (i.e., less exploration of the search landscape).

**Fig. 17:**
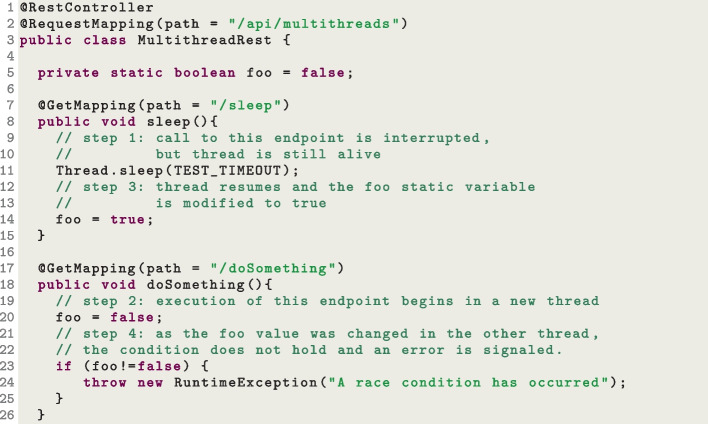
Snippet code of the class MultithreadRest, showing two HTTP endpoints (sleep and doSomething) leading to a race condition

Even if the right balance for *T* is chosen, still TCP timeouts are a major problem for white/grey-box test case generation tools. Many HTTP servers are multi-threaded, in which each incoming HTTP request is handled by a different thread. A timeout on the client making the HTTP call (e.g., EvoMaster) would not imply a timeout on the server. The server would still be processing the request on a thread. If the test generation tool then evaluates a new test case, it can well happen that the server is having two threads executing the requests (the previous one that is still running, and the new one). Figure 17 depicts this problem. Let us assume a first generated test case calls sleep. Since the test execution of the endpoint waits beyond the test case timeout, EvoMaster kills the invocation, but the server-side thread is still alive. In turn, EvoMaster makes a fresh HTTP call to doSomething, but when the condition is evaluated, as the first thread modified the variable foo to true, the condition fails, signaling the exception.

Similarly to foo, this can mess up all the data structures in the instrumentation, if those are not thread-safe. But, even when they are thread-safe, there is still the problem to properly trace each code execution to the right evaluated test. For example, if a new line code is reached, which evaluated test case was responsible for it? The previous timed-out test that is still running, or the new one? It can get tricky, especially when more than one timed-out HTTP call is still processing at the same time.

One possible solution to this problem that we have introduced in EvoMaster is the use of the so-called *kill-switch*. The idea is that we try to make sure that, once a test case evaluation is completed, no thread is left executing business logic code. To do this, we instruct the instrumentation runtime (“switch on”) to throw an exception as soon as any instrumentation method is called (e.g., when recording that a code line has been covered). Then, when we need to evaluate a new test case (or, more precisely, a new HTTP call, as a test case can be composed of several HTTP calls), we set the switch to off. By the time the new incoming HTTP is processed, there is a “high chance” that the previous timed-out one has been terminated already.

This approach does not guarantee to solve the problem in all possible cases, but it has worked well so far in all the SUTs we have applied EvoMaster on.

### Lessons learned

When dealing with the white-box fuzzing of real-world software, there are many complex engineering challenges to face. Providing usable solutions takes major engineering efforts, which not always can result in direct academic outputs. But those engineering challenges must be addressed if researchers want to provide solutions that can be used in practice and that can scale up to real-world systems. Having PhD students “re-inventing the wheel” each time for these engineering challenges is not a viable approach to push forward the boundaries of scientific research. This can also be a possible explanation why, among the several black-box fuzzers for Web APIs in the literature, EvoMaster is currently the only one that does white-box testing.

Sharing working solutions for these kinds of engineering challenges is something we do strongly support and recommend. However, the best way to achieve this goal in the software engineering research community is not straightforward, especially when considering academic output metrics.

## Common practices

In this section, we are going to discuss some basic software practices used in the development of EvoMaster. Those will likely be considered *trivial* by any experienced software engineer. However, as we found them critical in the development of a tool like EvoMaster, and considering the target audience of junior PhD students and postdocs, let us summarize them:

- You must use a version control tool, such as Git (others not so popular any more are SVN and Mercurial).
- Your tool prototypes are still software. Write tests for them.
- Use a Continuous Integration (CI) server, to make sure each new code commit does not break compilation, nor any existing regression test (see previous point). There are a few free CI providers. But their state can change. For example, when we started with the development of EvoMaster, we used TravisCI, but then moved to CircleCI, and now GitHub Actions. A CI server can also be run locally (e.g., using tools like Jenkins). When using CI servers, it is also easy to build and check the tool in different environments (e.g., different LTS versions of the JDK such as 8 and 17, and operating systems such as Linux and Windows).
- CI servers can also be used for other important academic tasks, like automatically archiving code on Zenodo (an open-repository for long-term storage of scientific digital artifacts) at each new software release (this can be done at the moment for example by using GitHub WebHooks), e.g., Arcuri et al. (2022).
- Never directly commit to the master/main branch of the repository. All development should be done on *branches*, and Pull Requests (PRs) must be created before the code is merged into the master/main branch. PRs should not be merged if CI fails (e.g., due to failing tests).
- Each PR must be manually reviewed (typically by the senior members in the team) before merging, even when CI passes.
- Documentation is very important. If no one can use your tool prototype because there is no information on how to run it, how can impact on practice be achieved? Furthermore, in the long run, documentation is useful to quickly onboard new PhD students and postdocs that join your projects, as well as when collaborating with industrial partners. Besides writing documentation, we found particularly useful to create quick training videos as well.
- Use some (lightweight) management tool to assign and keep track of tasks. We use Trello.
- If you do not want to spend most of your time debugging obscure bugs, stop using *mutable static state* in your programs, and rather use a *dependency injection* framework. For example, in EvoMaster we use Guice.
- If several people (e.g., PhD students and postdocs) will work on the tool throughout the years, it might be advisable to use a *strongly typed* programming language (e.g., Kotlin, Java and C# are good options).
- Whatever coding rules you decide to apply in your projects, write them down in a text document, added to the repository of the tool itself. This can help bootstrapping new members joining the team, as we do for EvoMaster^19^.

## Open-source community

### Problem definition

Besides publishing papers, it can be useful to release the implemented software prototypes to the public. This can be the case when the prototypes are mature enough to be used by practitioners, as well as to enable other researchers to replicate published studies. However, there are *legal* issues related to how to *release* a software prototype, and not all researchers might be aware of these legal technicalities.

### Why open-source

Since its inception in 2016, EvoMaster has been open-source. There were several good arguments behind such a choice, including boosting the impact of publicly funded research to practice, as well as to simplify replicated studies and extensions from third parties (which can increase academic metrics such as “citation count”). On the other hand, there are also some possible “worries” about making the choice of releasing an academic prototype as open-source. One is about losing possible “low-hanging fruits”, i.e., other research groups could use such prototypes to publish results that would not require too much work, and so lose such possible “easy” publications. Another possible issue is that a prototype could have some serious fault which invalidates most of its published results. Or some of its parts were implemented “ad hoc” for the specific case studies used in its empirical analyses. If the code is not open-source, hardly anyone would find out. And any failed replicated experiment from a third party could be attributed to just lack of details (as hardly any published scientific paper provides enough details in its text to enable a full experiment replication). In some cases publishing as open-source is not an option at all, e.g., when authors do not work for a public university or research institute, or depending on the source of funding of the research.

Regardless of whether to publish a research prototype as open-source or not, *replicability* is an important goal for the advancement of scientific research. Many venues (typical top-level conferences, but not journals, at least at the time of this writing) in Software Engineering ask for (but not require) “replication packages”. An example of this can be found in the *Open Science Policies* of ICSE^20^. Even if a tool is not released as open-source, at least it should be provided as an executable, to enable replicated experiments.

### Choice of license for EvoMaster

When publishing a research prototype as open-source, the first question is *which license to use?* There are several licenses to choose from, each one with its own benefits and negative sides. For the researchers that do not want to dig into all the nitty-gritty legal details of software licenses, choosing the right license can be a daunting task. It is not in the goals of this paper to provide a full detailed analysis of all open-source software licenses that are available. We are simply going to discuss why we chose a specific license for EvoMaster, explaining the reasoning behind our decisions. Although this is done with the aim of helping our fellow researchers that are in a similar situation, we would like to point out that we are Software Engineering academics, and not lawyers. What presented next does not constitute legal advice.

For EvoMaster, we chose the *GNU Lesser General Public License v3.0* (LGPL). We wanted practitioners to *use* EvoMaster, researchers to *extend* it for scientific reasons (e.g., for publishing their research, like Stallenberg et al. (2021)), but not for commercial actors to extend it as closed-source (which would be doable when less restrictive open-source licenses are used). As academics working in Software Engineering research, to improve impact of our research, we do collaborations with industry (Garousi et al., 2019). Using a more restrictive license like GPL would prevent/hinder this (e.g., due to what in the grey literature is often referred to as the “GPL Poisoning” effect), whereas most other open-source licenses (including LGPL) would be “fine”. This also impacted the development of EvoMaster, as we need to make sure to never add a GPL library to it (unless it has a “classpath exception” in its license), as it would “poison” the entire codebase, making it all GPL.

Regardless of the chosen license, the license can be changed afterwards (but not retroactively). Also, the same software can be released with different licenses at the same time. For example, we could make the next EvoMaster ’s release with a permissive Apache License, as well as providing all previous versions of EvoMaster with a GPL license. In this latter case, those existing versions would have 2 licenses, LGPL and GPL, although we would have no legal obligation of keeping distributing the versions licensed with LGPL. How a software is licensed depends on its *copyright holders*, and all copyright holders must agree on the chosen license. This has some very practical consequences. For example, we would not be able to make a new release of EvoMaster tomorrow with a different license (e.g., Apache) unless every single contributor agrees, as we do not own the copyright of the software they wrote. The alternative would be to remove every single line they wrote, and re-implement from scratch that functionality. To complicate the matter even more, a code contributor might not own the copyright of the software he or she developed. It might belong to their employer, which is typically the case for engineers in industry developing software during working hours using the employer’s hardware. Technically, this can also be the case for academics working at universities and research institutes. It all depends on the details of the job contracts they signed, and local laws. And there can be huge differences from country to country. This can be particularly troublesome for PhD students, as it is not uncommon that in some countries and institutes students (not just PhD) are required to sign away the copyright of all software they develop as part of their studies. The reasoning behind this is not necessarily malice, but bureaucratic simplification, e.g., to avoid disgruntled students sue their university for copyright infringement each time their software is copied from one machine to another (e.g., when the lecturers download the students’ software exams on their computer for marking). Technically, there are many examples of possible legal issues with coping and storing students’ software, especially when all exams have to be stored for a certain amount of years for legal reasons.

Even if one does not own the copyright of the software prototypes they develop, this is not necessarily an issue (at least, from an academic point of view). As long as the actual copyright owners (which could be a university and not the academic researchers) agreed to release the software as open-source, one can continue afterwards to work and extend such software using the *same* open-source license. You do not need to own the copyright of a software project to fork it and extend it. In recent years, one of the most known cases is when Amazon forked the open-source ElasticSearch in 2021 (and renamed it into OpenSearch), due to a change of license in the newer versions of ElasticSearch. The same concept applies when a research moves from one institute to another (e.g., in a different country). As long as the software was released as open-source, it does not matter much if he or she was owning the copyright when employed in the former institute, as long as there is no plan to change the license.

Regarding the choice of an open-source license, it needs to be done carefully. It can be changed afterwards, but such process can be complicated (for all the reasons we discussed). We use LGPL, but many of the other licenses would do as well. We just want to give a warning if someone chooses to use GPL, in case they want to do research collaborations with industry. Also, the use of open-source licenses helps with possible issues with copyright ownership. It can help to have research institutes/universities to agree *in writing* to open-source such research prototypes. A place we found useful, to have it in, is in research grant applications, where it can be specified that the output of the projects will be released as open-source. Note that all these legal issues are not just theoretical, as we unfortunately had to deal with some of them during the development of EvoMaster.

### Challenges of releasing as open-source

If one decides to release a prototype as open-source, as we did, it is important to clarify that putting the code on a repository such as GitHub is just the *beginning*. If the tool is addressing a *real problem* in Software Engineering practice, people in industry will start to use such a tool. And tools have faults. Engineers will use these tools on their systems, and those systems could have features/properties not present in any of the case studies used by the authors of those tools in their empirical experiments. In such cases, practitioners might start to report *issues*, i.e., write bug reports.

Whether to engage the practitioner community in fixing those reported issues is not something we can blindly recommend without reservations. It takes time. And often the required technical effort does not directly translate to something that can be publishable in an academic venue. Often, it is just technical work. And, as such issues are not present in the case studies used for experiments (otherwise they would have already been found and fixed), there is no immediate, concrete benefit from an academic standpoint.

But, there are benefits, in the *long* run. Fixing faults make a tool more *robust*, which helps when increasing the size of the selected case studies for new experiments (e.g., the corpus of web services we collected in EMB (Evomaster benchmark (emb), 2023) has been expanding throughout the years). It also benefits when the tool is used in tool comparisons, as it is less likely to crash on a new SUT. Let us make a concrete example to clarify this point. In our recent work (Zhang et al., 2022), we compared EvoMaster with two other fuzzers, namely RESTler (Atlidakis et al., 2019) and RestTestGen (Viglianisi et al., 2020), on five APIs running on NodeJS. For those APIs, these tools had bugs that hindered their applicability (e.g., on some of those SUTs, these tools just crashed). This is not completely fair, as in EvoMaster we fix all its faults when we start to use a new SUT for experimentation. For a third party, it could be possible to find some new SUTs for which EvoMaster crashes due to faults whereas RESTler and RestTestGen do not. This is also one of the main reasons to create EMB (Evomaster benchmark (emb), 2023), to enable a common set of SUTs that different authors can make sure their tools work on. Ideally, tool comparisons should be handled by third parties on an unbiased selection of SUTs. This is for example done for unit test generation of Java software in the SBST Tool Competition (Panichella et al., 2021). But these kinds of competitions are not so common, at least in the Software Engineering research community.

At the time of this writing, 80 issues have been reported on the GitHub repository of EvoMaster^21^. 74 of them have been resolved, and closed. Some of them are *feature requests*, like supporting test output formats such as Postman^22^ and Spring WebTestClient^23^. But the majority of these reported issues are simply crash reports, like for example #412^24^. However, so far no Pull Request (PR) has been made by practitioners (all PRs so far have been originated from the developers of EvoMaster and from academic collaborators).

### Lessons learned

As a rule of thumb, in most cases we recommend to publish research prototypes as open-source, using for example the *GNU Lesser General Public License* (LGPL) license. To avoid some categories of legal issues regarding copyright, we recommend to specify the use of open-source licenses in the writing of research grant proposals. Even if a researcher does not want to support and maintain an open-source prototype in the long run, other researchers can benefit from the released software.

There might be cases in which other licenses and release models could be more appropriate, but those require a deep understanding of all the possible legal issues involved.

## Generalization of lessons learned

This paper reports on our experience in developing EvoMaster over the last six years (at the time of this writing, in 2022). Such experience builds on top of our^25^ previous experience of developing EvoSuite, in the last 13 years. We believe that many of the lessons learned that we share in this paper can be of use for researchers in the Software Engineering research community, and not just in software testing. However, without empirical experimentation and future experience reports from other researchers, how well these lessons learned can generalize to the development of other research tools is hard to quantify in an unbiased way. Interviews and questionnaires with the developers of other successful tools (e.g., AFL and KLEE) could provide interesting insights.

Many of the lessons learned shared in this experience report are of very practical nature. As research work in Software **Engineering**, many details are low level, including source code examples, when needed. There is a trade-off between high-level, academic philosophical discussions that are more general, and low-level engineering details that might become outdated within the next 3–5 years. General philosophical discussions might have a better chance at surviving the *test of time*, but we still need to solve the software engineering challenges of today, right now. In this paper, we mention a few tools. This is done to put this work into concrete engineering ground, with actionable takeaways for the readers, but we would not be surprised if several of these tools will disappear in the future. But it does not matter if you use Trello instead of another tool, as long as you use a management tool to keep track of development tasks. At the time of this writing, some tools have been around for more than 25 years (e.g., SQL databases like Postgres and MySQL). However, this does necessarily imply that they will still be used 25 years from now^26^. On the one hand, many of the lessons learned we share in this paper would have been applicable and usable in the past. For example, a report like this would have saved us a lot of time and prevented many mistakes when in 2010 some of us were working on EvoSuite. Also, dealing with resetting SQL databases for testing purposes is something that has been an issue for decades. It is just not much research work was done on *system* test case generation for enterprise/web applications. Furthermore, the lesson of putting effort into fully automating a data analysis pipeline (e.g., from experiments to automatically generated tables/graphs automatically imported into Latex papers) is something that, technically, could had been already put into practice 20–30 years ago (Latex is from 1984, whereas R is from 1993). On the other hand, there is no guarantee that the lessons learned shared in this paper will be still relevant 20 years from now. But, for an engineering discipline, we do not consider this as a major threat to validity.

Some of the insight shared in this paper is specific for prototypes developed in academia (e.g., dealing with all the scaffolding for running experiments), whereas others are very general (e.g., the importance of Continuous Integration). General guidelines and best practices for software development have been already extensively reported in the grey literature, and likely as well in modern textbooks in software engineering. We do not claim those as a novel contribution in this paper. We simply report on what had a major impact in the development of EvoMaster, to make sure that readers have an easy access to at least a summary of key points that are likely going to be useful for the development of their research prototypes.

## Conclusion

In this paper, we have reported on our experience in building the EvoMaster test case generator tool, over the last six years. We discussed how to simplify the running of experiments, and how to simplify the collection and automated analyses of the results. We also provided concrete solutions to several technical challenges in the implementation and use of this kind of research prototype.

Our goal with this experience report is to inspire and help bootstrapping new development effort in the Software Engineering research community. Ultimately, such effort should help close the gap between academic research and industrial practice.

EvoMaster is stored on both GitHub and Zenodo. To learn more about EvoMaster, see our website: www.evomaster.org

## Data Availability

All data used to conduct this study (i.e., EvoMaster source code, E2E tests and examples of scripts for running experiments and analyzing experimental results) is available on both GitHub (www.evomaster.org) and Zenodo (Arcuri et al., 2022).

## Footnotes

1 https://github.com/google/AFL

2 https://github.com/klee/klee

3 https://github.com/EvoSuite/evosuite

4 https://github.com/randoop/randoop

5 https://github.com/EMResearch/EvoMaster

6 https://github.com/EMResearch/EvoMaster/graphs/contributors

7 GitHub stars might be useful as a *metric* only as long as they do not become a *target*, as it is not so difficult to maliciously inflate their number

8 The driver for this example can be found in https://github.com/EMResearch/EMB/blob/master/jdk_8_maven/em/embedded/rest/scs/src/main/java/em/embedded/org/restscs/EmbeddedEvoMasterController.java

9 https://github.com/rest-assured/rest-assured

10 https://github.com/jopt-simple/jopt-simple

11 https://github.com/EMResearch/EvoMaster/blob/master/docs/options.md

12 https://www.sigma2.no/

13 https://www.docker.com

14 https://github.com/EMResearch/EvoMaster/blob/master/docs/replicating_studies.md

15 https://github.com/EMResearch/EvoMaster/blob/master/docs/publications.md

16 https://github.com/EMResearch/EvoMaster/actions

17 https://www.testcontainers.org

18 https://eur-lex.europa.eu/eli/reg/2016/679/oj

19 https://github.com/EMResearch/EvoMaster/blob/master/docs/for_developers.md

20 https://conf.researchr.org/track/icse-2022/icse-2022-open-science-policies

21 https://github.com/EMResearch/EvoMaster/issues?q=is%3Aissue++

22 https://github.com/EMResearch/EvoMaster/issues/260

23 https://github.com/EMResearch/EvoMaster/issues/227

24 https://github.com/EMResearch/EvoMaster/issues/412

25 Two of the authors of EvoMaster were among the main contributors of EvoSuite

26 Although still having to deal with SQL Injection in the 24th century does not sound so improbable (Star Trek Discovery, “If Memory Serves”, 2019)
